# Congenital Hypoplasia of the C1 Posterior Arch Masquerading as Multiple Sclerosis: A Case Report

**DOI:** 10.7759/cureus.118319

**Published:** 2026-10-21

**Authors:** Youssef A Abdelmegeid, Ikinu Wambugu, Mohamed M Hegazy, Mahmoud AlHadidi

**Affiliations:** 1 Medicine and Surgery, Manchester University NHS Foundation Trust, Manchester, GBR; 2 Medicine and Surgery, Salford Royal Hospital, Manchester, GBR; 3 Orthopaedics and Trauma, Ahmadi Hospital-Kuwait Oil Company, Al Ahmadi, KWT

**Keywords:** c1 hypoplasia, cervical myelopathy, currarino classification, multiple sclerosis, myelomalacia

## Abstract

Hypoplasia of the C1 posterior arch is a rare congenital anomaly, classified using the Currarino system into subtypes A through E depending on the extent and nature of arch deficiency. The majority of cases are incidental radiological findings; symptomatic presentations causing cervical myelopathy are exceptionally uncommon and not well represented in the surgical literature. In such cases, the absence of established management guidelines renders clinical decision-making particularly challenging.

We present a case of a 45-year-old male surgeon who presented with a one-year history of episodic bilateral upper and lower limb weakness. Initial investigations, including brain and cervical spine MRI, were interpreted as consistent with a diagnosis of multiple sclerosis, for which treatment was commenced. Following a symptom-free interval after a one-month course of corticosteroids, the patient experienced a recurrence at eight months, prompting dynamic MRI of the cervical spine, which revealed a type C hypoplastic C1 posterior arch causing dynamic spinal cord compression during hyperextension. The diagnosis was revised accordingly. Supplementary investigations confirmed atlantoaxial stability and normal vertebral artery anatomy bilaterally. The patient provided consent and underwent posterior surgical decompression of the entire posterior arch, without instrumented fusion. Intraoperative neurophysiological monitoring guided the extent of resection; decompression began on the fibrous left-sided arch, extending to the level of the vertebral artery groove, followed by the bony right-sided arch, sequenced to preserve facet joint integrity. The patient achieved complete symptom resolution postoperatively.

This case highlights the diagnostic challenge posed by symptomatic C1 arch hypoplasia, which may mimic demyelinating disease, and demonstrates that surgical decompression, guided by intraoperative neurophysiological monitoring and tailored to arch morphology, can achieve excellent neurological outcomes without the need for instrumented stabilisation.

## Introduction

The collapse of an accomplished surgeon in theatre, due to a congenital anomaly manifesting at 45 years of age, is a sentinel event we must all learn from. Abnormalities affecting the craniovertebral junction are rare but clinically significant. These anomalies arise from incomplete chondrogenesis and result in a range of malformations, from minor vertebral clefts to complete absence of the posterior C1 arch [1].

The atlas (C1) is the uppermost cervical vertebra and forms a ring around the upper cervical spinal cord. It consists of anterior and posterior arches connected by paired lateral masses. The posterior arch normally forms a continuous bony structure behind the spinal cord. Congenital defects of this arch are uncommon developmental anomalies arising from abnormal formation and fusion of the posterior elements of C1 [1].

These abnormalities are commonly described using the Currarino classification [2]. The anomaly relevant to this case, Currarino type C, consists of bilateral defects of the posterior arch with preservation of a posterior osseous component. This retained posterior element is clinically important because abnormal movement during cervical extension can narrow the available spinal canal and dynamically compress the underlying spinal cord [2]. Patients may develop progressive or episodic limb weakness, sensory disturbance, fatigue, and other manifestations of cervical myelopathy.

The reported incidence of posterior arch anomalies of the atlas ranges from 0.69% to 2.95% [1,3], with the majority discovered incidentally on imaging performed for other indications [3]. Although significant when symptomatic, cases presenting with myelopathy are rare, and those resulting in a diagnosis of multiple sclerosis represent only a handful of published cases globally [4-6].

Clinical and radiological overlap can therefore create diagnostic uncertainty between compressive myelopathy and demyelinating disease. In this case, this overlap led to C1 arch hypoplasia being misdiagnosed as multiple sclerosis [7,8].

Diagnosis of Currarino type C has proved challenging, as compression of the spinal cord by the compromised posterior arch can only be seen in a position of dynamic neck extension [4]. Currently, standard imaging, including MRI, is usually acquired in a neutral position and therefore cannot demonstrate position-dependent compression, subsequently leading to failure to identify this anomaly [9]. Correct diagnosis requires dynamic imaging modalities for definitive identification and characterisation [4,9].

We present a rare case of myelomalacia secondary to Currarino type C posterior C1 arch hypoplasia that was initially interpreted and treated as multiple sclerosis. The case illustrates the potential diagnostic overlap between demyelinating and dynamic compressive cervical cord disease.

## Case presentation

We report a case where a 45-year-old male presented to a neurophysician’s clinic after struggling for a year with symptoms of sudden-onset upper and lower limb weakness with no sensory deficit, no clear precipitant, preserved consciousness, and no other associated symptoms such as pain, and recovering within a few minutes with no symptoms between attacks. These symptoms fit the paroxysmal neurological symptoms that some multiple sclerosis patients first present with [10]. The patient attributed these symptoms to generalised fatigue. The patient is a surgeon himself, and what triggered him to seek medical attention was the acute onset of bilateral upper and lower limb weakness, causing him to collapse during surgery.

At the neurologist's clinic, the initial examination findings were unremarkable as the examination was conducted after the bout of weakness had ended. Thus, upper and lower limb power, tone, sensation, gait, co-ordination, and reflexes were normal. Given the episodic nature of the symptoms, a brain and C-spine MRI was performed. The investigations revealed a small intramedullary focus in the cervical cord (Figure 1), and the brain MRI showed tiny bilateral juxtacortical cerebral foci with altered signal. Based on episodic upper and lower limb weakness and the concurrent radiologist report, a working diagnosis of multiple sclerosis was made, and a one-month course of prednisolone was initiated, starting at 200 mg daily for seven days, which was then tapered down to 80 mg daily for the remaining three weeks. No immunomodulators were initiated as the patient had no further symptoms after the first month of treatment. Eight months after starting treatment, another bout of weakness occurred, prompting him to see the neurologist again.

**Figure 1:**
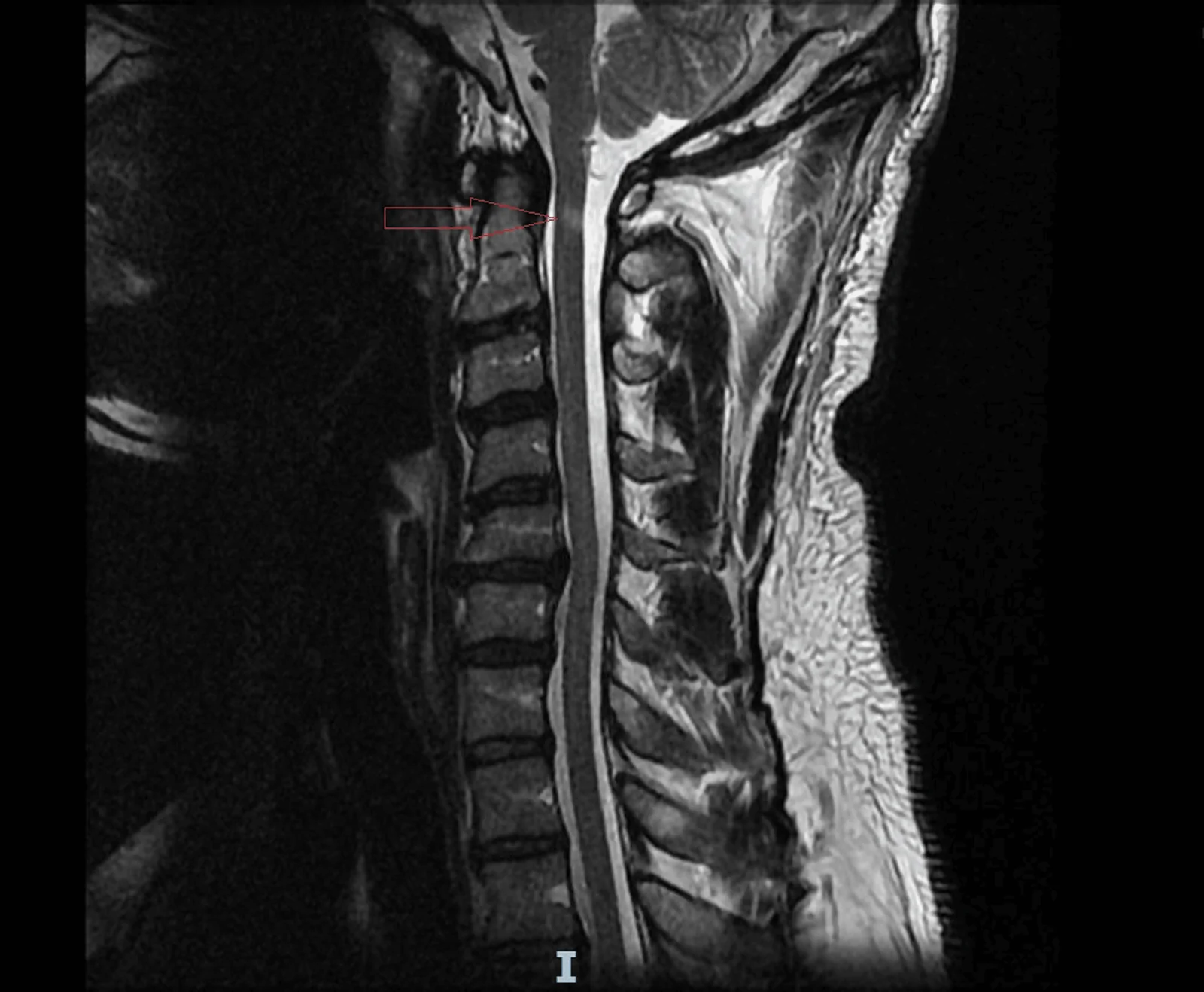
Sagittal image of a static MRI in a neutral neck position. Arrow pointing towards T2-weighted hyperintensity.

Subsequently, diagnostic reassessment was prompted by the recurrence of episodic weakness eight months after starting corticosteroid therapy. In the context of recurrent attacks, negative cerebrospinal fluid oligoclonal bands on lumbar puncture at reassessment, and a known cervical cord signal abnormality, a structural cause was considered.

On review of the history, the positional nature of the symptoms was noted, as the initial collapse had occurred while the patient was operating in theatre. To test the possibility of a structural, position-dependent cause, a dynamic cervical MRI was performed. This demonstrated position-dependent compression of the spinal cord at C1 during hyperextension, caused by a Currarino type C posterior arch abnormality (Figure 2). In light of these findings, the symptom-free interval following corticosteroid therapy was considered likely to be coincidental, and the patient was referred to the spinal orthopaedic team.

**Figure 2:**
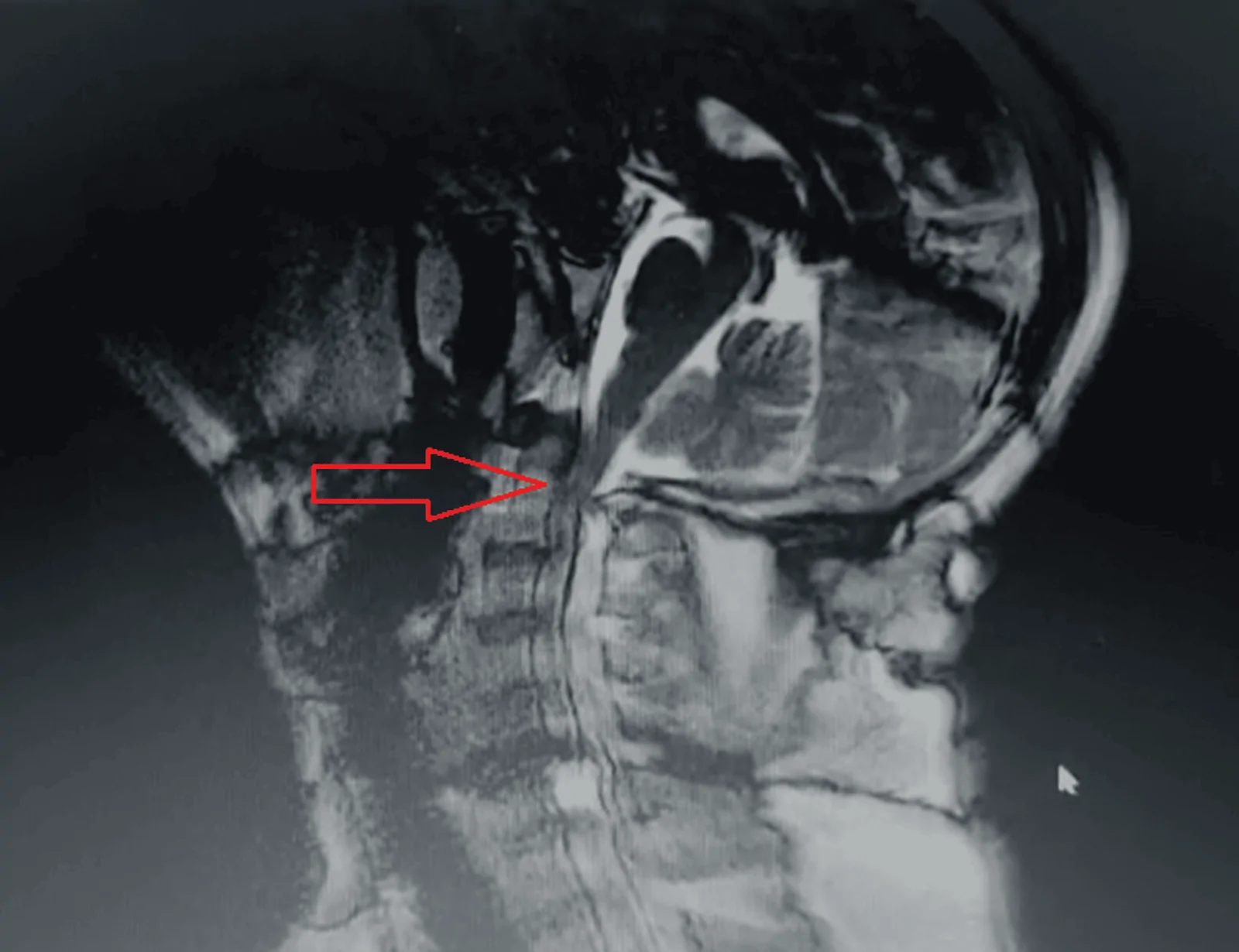
Dynamic MRI of the neck in extension. Sagittal image of the neck in extension during dynamic MRI with an arrow showing compression of the spinal cord.

We then had a frank discussion about the findings and that the cause of his myelomalacia was as a result of hypoplasia of his C1 arch, which is likely congenital in nature. We explained that on rare occasions, this hypoplasia can be symptomatic. We discussed that the treatment of this would be surgical, and it would involve decompressing the spinal cord and removing the part compressing the cord; we also discussed a plan for the surgery. Flexion-extension radiographs of the cervical spine were obtained showing no evidence of atlantoaxial instability. We performed a CT C-spine (Figure 3) to classify the type of C1 arch hypoplasia (type C) using the Currarino classification. Moreover, a vertebral artery CT angiogram showed that the arteries were normal bilaterally (Figure 4). These findings were discussed with the patient, and he agreed to a surgical intervention to decompress the spinal cord at C1. We also clarified that we could not guarantee that surgical decompression would alleviate symptoms and that there was also a risk of infection, pulmonary embolism, C-spine instability, and injury to the spinal cord, which could lead to quadriplegia.

**Figure 3:**
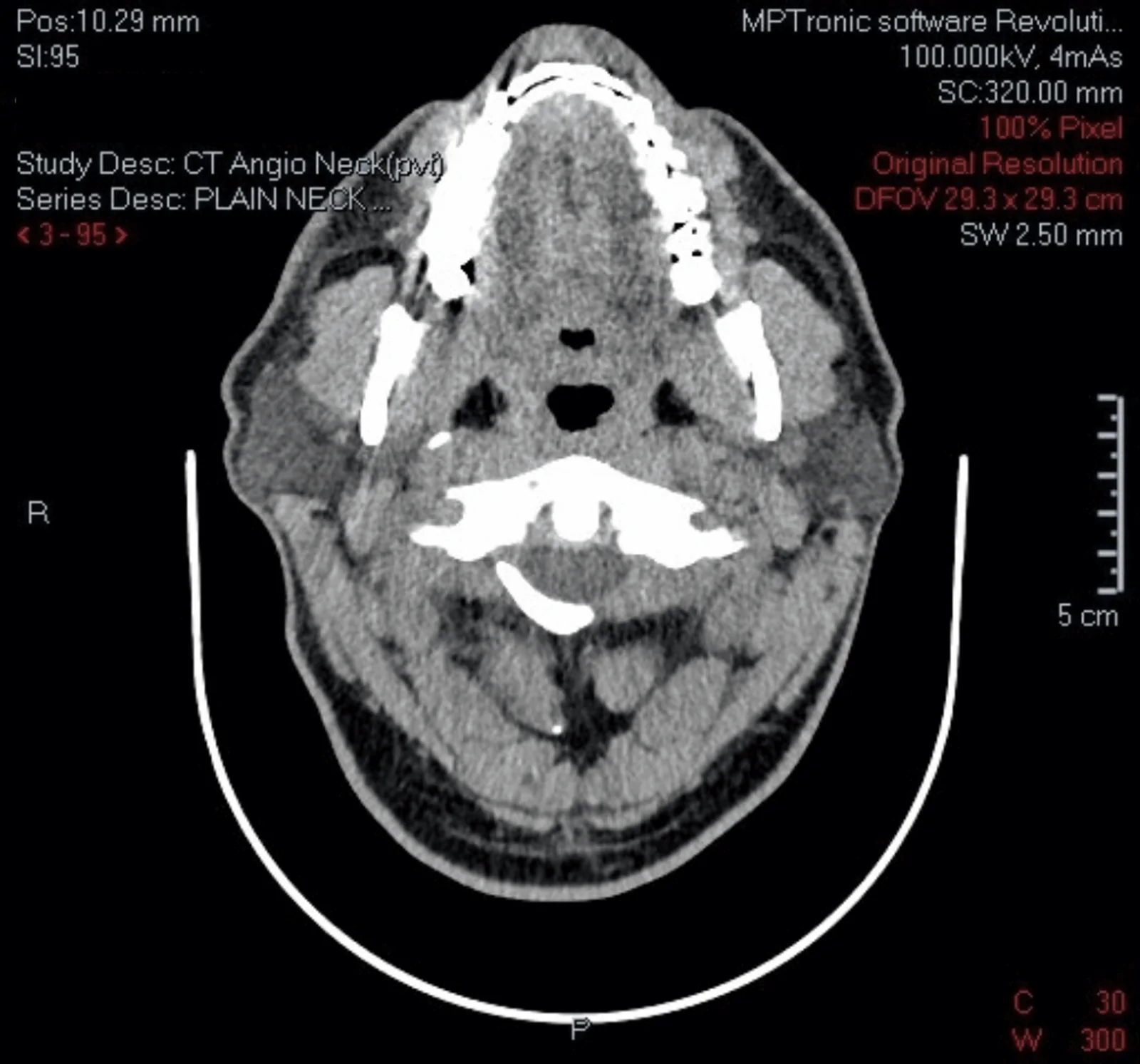
Axial CT of C1 vertebrae. This image shows the left fibrous side and the mostly bony right side with a small fibrous part of the C1 vertebrae.

**Figure 4:**
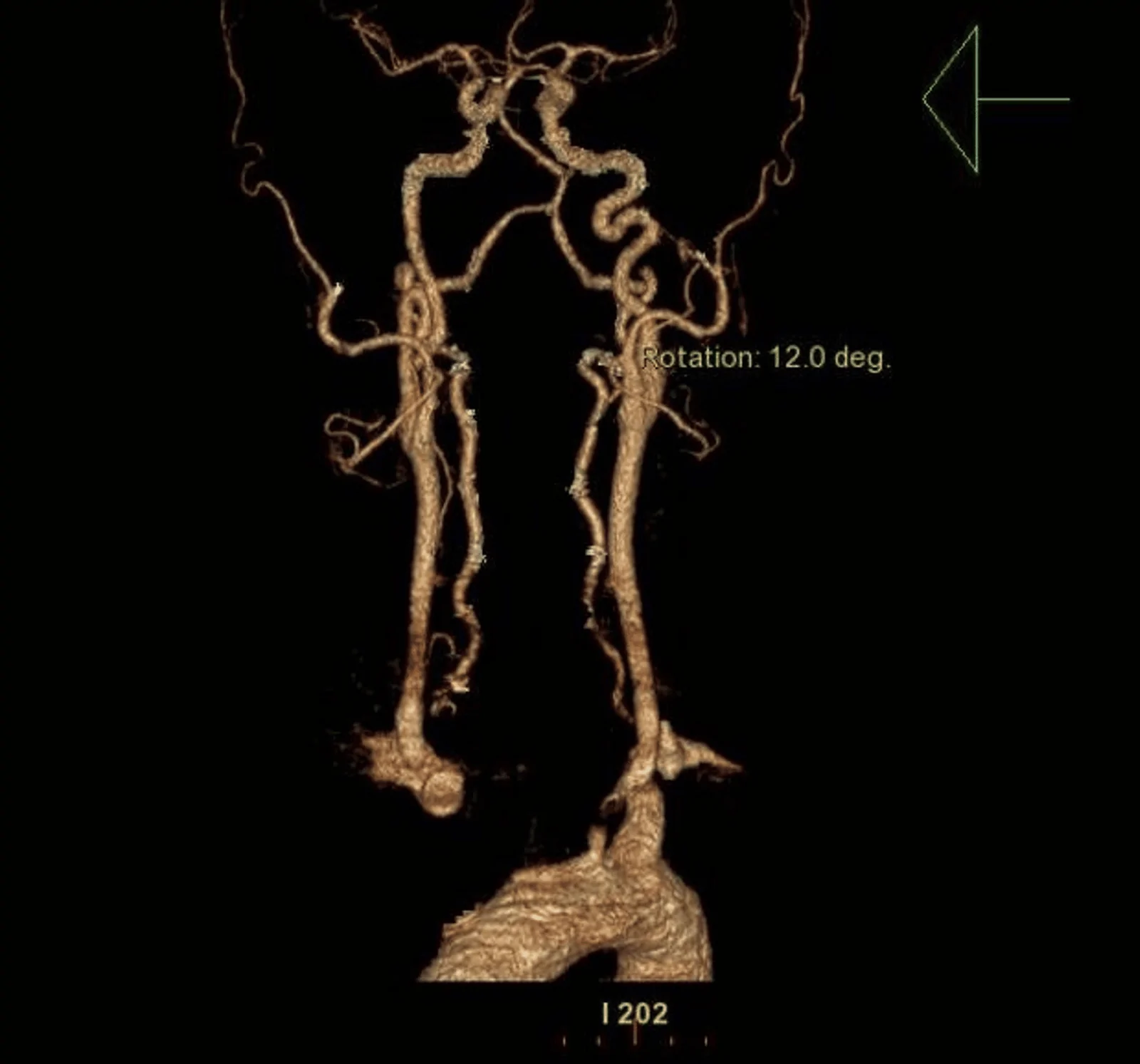
CT angiogram of the vertebral arteries in the neck showing normal anatomy. CT angiogram showing no compromise of the blood supply.

The patient underwent surgical posterior decompression via excision of the hypoplastic C1 posterior arch elements. Given the absence of atlantoaxial instability and preservation of bilateral facet joint integrity, instrumented fusion was not performed. Under general anaesthesia, the patient was positioned prone with the head secured in a Mayfield clamp using temporal pin fixation. Intraoperative neurophysiological monitoring was employed throughout the procedure. The C1 level was localised fluoroscopically under C-arm guidance and marked before incision.

A posterior midline incision was made through the ligamentum nuchae. A stay suture was placed on the right paraspinal musculature to maintain exposure. Subperiosteal dissection revealed the C1 posterior arch, which was found to be adherent to the underlying neural structures. Under microscopic magnification, the arch was carefully identified and partially released along its medial aspect. Notably, the left-sided arch was fibrous in consistency, while the right-sided arch was mostly bony in nature, except for a small fibrous part (Figure 3).

The entire posterior arch was resected until reaching the vertebral arteries on both sides. Attempts to section the fibrous tissue on the left side were initially undertaken using conventional instrumentation; however, these were suspended after changes in intraoperative neurophysiological monitoring signals, indicating cord compression. Accordingly, an ultrasonic bone scalpel (Misonix, Farmingdale, NY) was employed, permitting a controlled cut of a small bony part along the midline. This facilitated wide retraction of the fibrous band and exposure of the dura and spinal canal. Surgical dissection was then continued on the left side, with progressive removal of residual fibrous tissue, extending laterally to the level of the vertebral artery groove. Decompression of the right-sided bony arch was also done after the left fibrous side to avoid compromise of facet joint integrity and the potential need for supplementary instrumented stabilisation.

The wound was closed in layers over a suction drain, and the post-operative period was uneventful with a soft cervical collar applied. The patient was followed up and clinically examined after six months and 24 months, with unremarkable neurological findings in tone, power, sensation, co-ordination, reflexes, and gait. The patient had no further episodes of weakness; thus, no further imaging was done. There were no post-surgical complications.

## Discussion

The principal lesson of the case presented here is one of diagnostic awareness. The symptom complex of C1 arch hypoplasia myelopathy is episodic limb weakness and intramedullary T2 signal change; this closely mimics demyelinating disease, and the initial misdiagnosis of multiple sclerosis in this case is a pattern documented in the prior literature [7,8]. The discriminating investigation was dynamic MRI, which demonstrated compression of the cord at C1 exclusively during cervical hyperextension. This finding is invisible on static imaging acquired in a neutral position. This is the fundamental biomechanical characteristic of type C hypoplasia: the pathological arch compresses the cord only in hyperextension, and standard MRI will reliably miss it [4,9].

The diagnostic value of dynamic imaging in this case arose from the recurrence of symptoms after corticosteroid therapy, combined with a diminishing index of suspicion for multiple sclerosis following negative oligoclonal bands. Dynamic MRI is not a routine investigation in suspected demyelinating disease, but it may provide valuable targeted information when patients have negative CSF findings or fail to respond to treatment. It may provide a valuable targeted investigation when clinical or radiological features specifically suggest position-dependent cervical cord compression, such as neurological symptoms reproducibly associated with neck movement or discordance between symptoms and neutral-position imaging (Figure 1). In the present case, extension imaging demonstrated posterior C1 cord compression that was not appreciated on the initial static studies.

Following decompression, the patient experienced complete resolution of symptoms. The outcome therefore supports successful relief of the dynamic mechanical compression rather than reversal of established myelomalacia.

## Conclusions

This case highlights the value of recognising clinical-radiological discordance. When symptoms and examination findings are not adequately explained by static imaging, the diagnosis should be revisited rather than accepted on the basis of reassuring scans. In selected patients where a position-dependent mechanism is suspected, dynamic imaging may reveal pathology that conventional studies miss. Here, identification of the underlying structural cause led to surgical decompression and a successful neurological outcome. Although conclusions from a single case are necessarily limited, it underlines the importance of maintaining a broad differential that spans specialities such as neurology and orthopaedics.
